# The Effect of Rare Earths Additions on the Microstructure and the Corrosion Behavior of Sn-0.7Cu-0.075Al Solder Alloy

**DOI:** 10.3390/ma12223731

**Published:** 2019-11-12

**Authors:** Wenchao Yang, Zaixiang Du, Shuyuan Yu, Yitai Li, Junli Feng, Xuanchen Wei, Qiang Li, Yongzhong Zhan

**Affiliations:** 1 School of Resources, Environment and Materials, Guangxi University, Nanning 530004, China; ywch053@163.com (W.Y.); imzx_to@126.com (Z.D.); xuanchen_wei@163.com (X.W.); xutaofromyjt@163.com (Q.L.); 2 Guangxi Key Laboratory of Processing for Non-ferrous Metal and Featured Materials, Nanning 530004, China; 3 Shenzhen Customs Industrial Products Inspection Technology Center, Shenzhen 518067, China; szciqysy@126.com (S.Y.); fjlhhp@foxmail.com (J.F.); 4 Shenzhen Academy of Inspection and Quarantine, Shenzhen 518010, China

**Keywords:** Sn-Cu-Al solder, rare earths, microstructure, corrosion

## Abstract

Sn-0.7Cu-0.075Al solder alloy adding with Ce and La had been successfully prepared by applying ball-milling and vacuum arc remelting. The influence of Ce and La on microstructure and corrosion behavior of Sn-0.7Cu-0.075Al solder alloy was investigated. The results showed that Ce (La)-containing solders had refined grains and obvious directional tendency due to the dispersive refiner (CeO_2_ and La_2_O_3_). Electrochemical potentiodynamic curves revealed three different stages of the reaction, including anodic and cathodic processes, prepassivation section, and stable passivation stages. The self-corrosion potential (E_corr_) of alloys with Ce and La addition were a little bit more negative, hardly making a difference on corrosion occurrence. However, the corrosion current density (I_corr_) and passivation current density (I_p_) decreased by two-thirds and one-half respectively, which indicated a better corrosion resistant after adding rare earths. The recorded micrographs of corroded surface at different polarized points witnessed the formation of corrosion product film both on prepassivation and passivation stage. Moreover, the cross section of corrosion product film showed the coarse, loose film in Sn-0.7Cu-0.075Al solder and adherent, compact film in Ce (La)-containing solders, which further indicated an excellent anti-corrosion property.

## 1. Introduction

Toxic Pb in Sn-Pb solder swarmed into the waste streams of electronic industries in the past years, and restrictive regulations have been worked to make wide usage of a variety of lead-free solders possible [1,2]. The near eutectic Sn-Cu alloy system is a suitable alternative due to its low cost and good mechanical properties [3,4]. Since lower brazing temperature is not necessary for wave soldering, Sn-Cu solders have widely come into commercial use in that area [5,6]. However, microelectronic equipment meets the requirements against corrosive media, as a result, protecting ecosystems and human health from dissolved heavy metal content should be put on the agenda [7,8]. Hence, the study of corrosion behavior of Sn-Cu alloys is imperative.

Limited attention is paid on the corrosion properties about Sn-Cu solders. Li et al. [9] examined the E_corr_ and I_P_ of Sn-Cu alloy and reported that its corrosion resistance was similar to Sn-Ag-Cu and Sn-Ag but better than Sn-Pb solder. Gao et al. [8] revealed the different corrosion behaviors between Sn-0.75Cu solder and Sn-0.75Cu/Cu joint with the former performed better. Some scientific research [10,11,12] found that intermetallics of Sn-Cu solders played an important role in the process of corrosion and their corrosion products. These corrosion products have been detected to evaluate their behaviors. Some studies focused on the whisker growth of Sn-Cu alloy in various corrosion environment. The results presented that the whisker growth direction and the whisker growth rate of Sn-Cu alloy were detected in 3.5% NaCl aq. added with high and low electric field [13], while copper-oxide whiskers could be observed to plated on copper substrates when exposed Sn-Cu alloy coatings to elevated temperature and humidity conditions [14].

Effect of rare earth (Ce and La) on microstructures and mechanical properties of lead-free solders has been summarized sufficiently [15,16,17,18,19,20,21,22,23,24,25]: the finer microstructure, higher tensile strength, and better solderability had been achieved in Sn-Ag-Cu, Sn-Ag, Sn-Zn, and Sn-Cu solders which included Ce or La. On the other hand, RE alloying had been proved to be an effective method of optimizing the corrosion performance of some steel, aluminum, and magnesium alloys [26,27], but rarely enough on lead-free solders in corrosion behavior. 

Recently, Yang et al. [28] reported that Sn-0.7Cu-0.075Al had superior mechanical properties due to the refined microstructure, the dispersed IMC and the uniform stress distribution. However, when it came to corrosion resistance, the directionally solidified method was used to prepare alloys with different microstructure arrays, and found that the corrosion resistance of Sn-Cu solder which characterized by coarser cellular microstructure array was better than the finest cellular microstructure, which was reported by Osório et al. [29,30,31]. Based on trace element aluminum leading to superior mechanical properties, the Sn-0.7Cu-0.075Al alloy was selected as the matrix alloy.

The main purpose of this work was to demonstrate the effect of the rare earth Ce and La on the electrochemical behaviors of the Sn-0.7Cu-0.075Al alloy, especially in corrosion potential, polarization behavior, and passivate phenomenon. At the end of this article, the mechanism through rare earth addition was discussed, which was based on the results of the experiment.

## 2. Experimental Procedure

### 2.1. Materials and Samples Preparation

The basic alloys Sn-0.7Cu-0.075Al (denoted as SCA in this paper) were prepared by commercial starting materials Sn (99.9%, 38 μm mean grain size), Cu (99.85%, 10 μm mean grain size), and Al (99.7%, 3 μm mean grain size) powders. In order to obtain uniform component of Sn-0.7Cu-0.075Al alloy, a procedure that solve the problem of strong repulsive force in both Al-Sn and Al-Cu binary system had been used. The elemental powders of nominal composition were mixed in a planetary ball mill (90 r/min, 20 min, BXQM-2L, Nanjing, China), and then were compacted to an ingot of 10 g at a pressure of 15 MPa each sample. Afterwards, the ingots added with Ce (>99.9 wt %) and La (>99.9 wt %) respectively were solution-treated and homogenized by melting in a vacuum arc remelting furnace for four times to produce Sn-0.7Cu-0.075Al-0.25Ce (SCAC), Sn-0.7Cu-0.075Al-0.25La (SCAL).

### 2.2. Microstructural Observation

Specimens were mounted in epoxy. The mounted specimens were grinded and polished according to standard metallographic techniques. The pregrinding processes of SCA, SCAC, and SCAL alloys were caused by waterproof abrasive papers with various degree of roughness from grit 1000 to grit 5000. A silk and a SiO_2_-containing polishing solution were used to polish without being etched because the SiO_2_-containing polishing solution was alkalescent. The microstructures were examined by optical microscope (OM, MDS400, Chongqing, China) and scanning electron microscopy (SEM, Hitachi S-3400, Tokyo, Japan) with the energy dispersive X-ray (EDX) spectrom was used to determine the rare earth compositions at selected areas. The phases of the alloy samples were identified using an X-ray diffraction (XRD, Rigaku D/Max 2500V, Tokyo, Japan) using CuKα radiation, operating at 40 KV, 200 mA and a scanning rate of 8 (°)/min at diffraction angle 2θ from 20 to 90°.

### 2.3. Electrochemical Corrosion

Before electrochemical potentiodynamic measurements, the samples were machined to be 10 × 10 × 5 mm using electric spark cutting, and ground to a 5000 grit surface finish using silicon carbide paper, followed by distilled water washing and air drying. Then the selected samples were positioned into a glass cell with a circular 1.0 ± 0.02 cm^2^ surface immersed in a naturally aerated and stagnant solution (250 mL) of 3.5 wt % NaCl at room temperature (25.0 ± 0.1 °C). A working electrode (i.e., samples), a platinum counter-electrode and a saturated calomel reference electrode (SCE) were designed to test the potentiodynamic polarization curves log I = ƒ(E) which were determined by a wide range from −900 mV to 1700 mV (vs. SCE), at the scan rate of 2 mV/s. The characterization of corrosion products, including the microstructure and the interface morphology, were investigated with a scanning electron microscopy (SEM, Hitachi S-3400, Tokyo, Japan), an energy dispersive spectroscopy (EDS) and a X-ray diffractometer (XRD, Rigaku D/Max 2500V, Tokyo, Japan) when polarized to different points marked at polarization curves. The corrosion rate is calculated by the following formula

(1) $$Corrosion\;rate=\frac{A\times I_{\mathrm{corr}}}{n\times F\times \rho }\times 87600$$

The unit of corrosion rate is mm/a; A—relative atomic weight of the sample; I_corr_—corrosion current density, its unit is A·cm^−2^; n—number of electron transfer in electrochemical reaction; F—constant, 26.8; *ρ*—density of alloy, its unit is g·cm^−3^.

## 3. Results and Discussion

### 3.1. Microstructural Characterization

**Figure 1:**
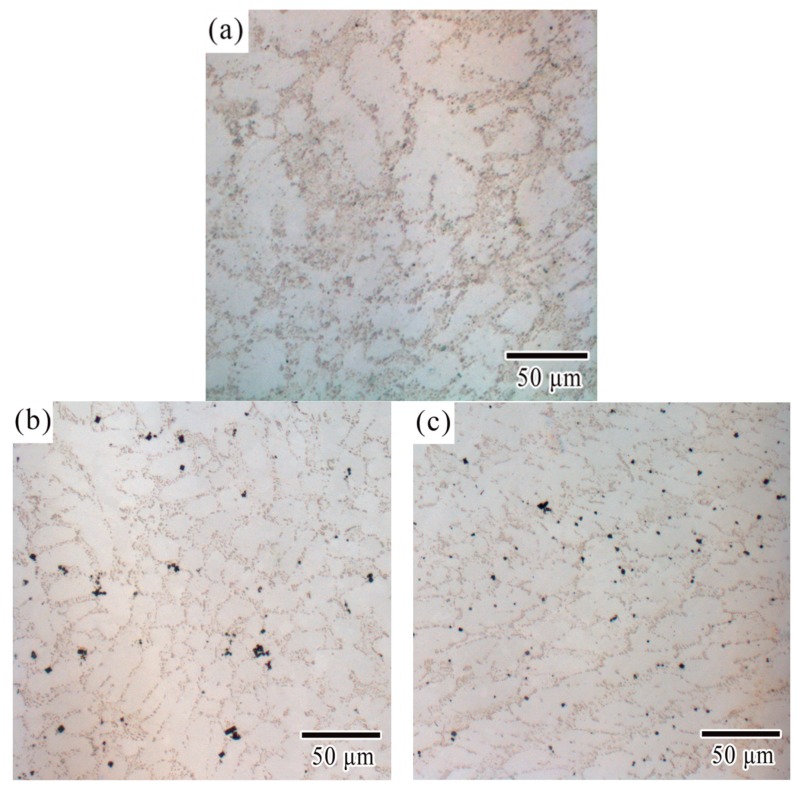
Microstructure of (**a**) SCA, (**b**) SCAC, and (**c**) SCAL solders by light microscopy.

**Figure 2:**
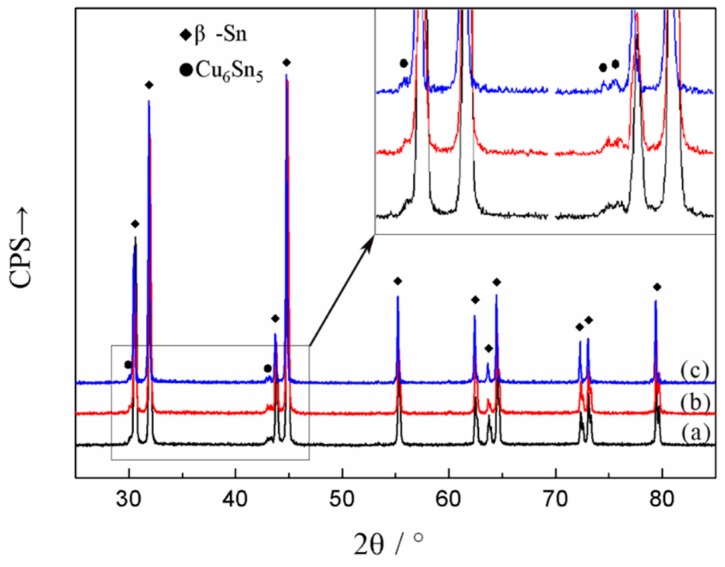
XRD diffraction patterns of (**a**) SCA, (**b**) SCAC, and (**c**) SCAL solders.

Figure 1 showed the microstructure of Sn-0.7Cu-0.075Al lead-free solders by light microscopy and the effects of adding cerium and lanthanum. As was shown in Figure 1a, the microstructure of Sn-0.7Cu-0.075Al solder consisted of a matrix single phase and eutectic structure. The XRD results in Figure 2 confirmed that the matrix single phase (bright region in Figure 1a) was β-Sn, and the eutectic structure (dark region in Figure 1a) was Cu_6_Sn_5_/Sn eutectic. This result matched those with the previous literature that researched on Sn-0.7Cu alloy [32,33,34]. As for Sn-0.7Cu alloy adding Al, the existence of Al depends on its content: the Al-Cu binary compound is formed preferentially according to thermodynamics; very little Al atom solid solutes in Sn matrix could be possible based on the Al-Sn phase diagram; another situation could be Al particles exist in Sn matrix as the reference [35] recorded. Yang et al. [28] noted that the IMC Cu_6_Sn_5_ in the Sn-0.7Cu alloy would partly transform into Al_2_Cu when aluminum content exceeded 0.05 wt %, but according to Lai et al. [36], the Al_2_Cu was not detected when aluminum content was under 0.15 wt %. In this study, when aluminum content was 0.075 wt %, the Al_2_Cu was not found by XRD, SEM and EDS technologies, and the reason could be that the trace Al atom solid soluted in Sn matrix.

**Figure 3:**
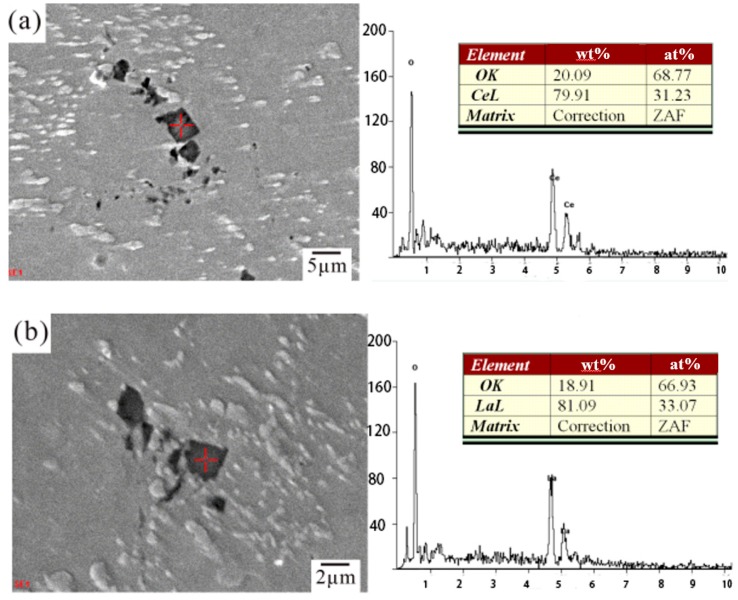
SEM images and EDS results of (**a**) SCAC and (**b**) SCAL solders.

According to Figure 1b,c, the changes of microstructure after the addition of Ce and La in the SCA alloy can be observed. With the addition of Ce, finer and more uniform β-Sn grains had been obtained, the refined microstructure of Cu_6_Sn_5_ particles had been formed, and the directional tendency of grain growth emerged, as illustrated in Figure 1b. The effects of adding La were similar with adding Ce. Furthermore, with the addition of La, the region between the β-Sn grain boundaries which mainly made up of distributed Cu_6_Sn_5_ phase became slender (see Figure 1c). This was because rare-earth-containing particles worked as grain refiner during solidification. Some of rare-earth contained particles—such as CeSn_3_, LaSn_3_, and CeO_2_—have been identified by other authors [18,19,20,21,22,23,37]. In this work, the square-like phase was speculated to be CeO_2_ by EDX/SEM, as illustrated in Figure 3a. The EDS result in Figure 3b confirmed the presence of oxygen and lanthanum, these square-like shape particles were identified to be La_2_O_3_.

### 3.2. Electrochemical Corrosion Analysis

**Figure 4:**
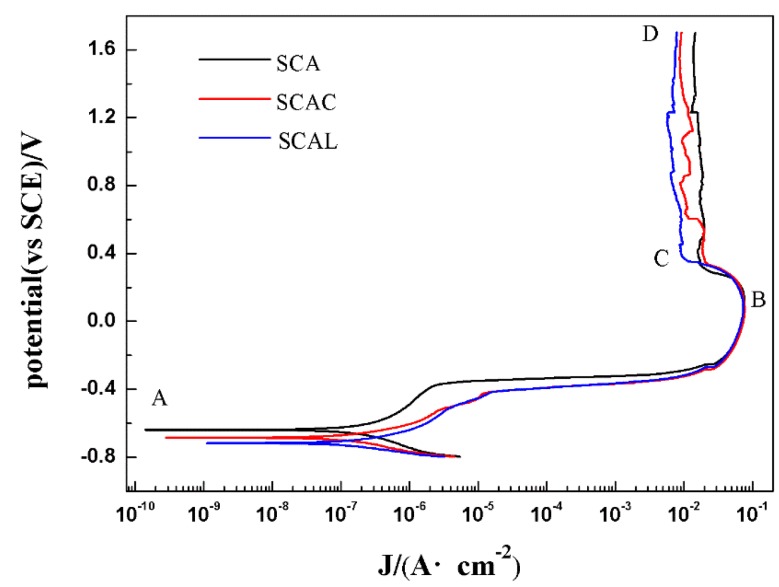
Polarization curves of SCA, SCAC, and SCAL solders in 3.5% NaCl.

The potentiodynamic polarization curves of SCA, SCAC, and SCAL solder samples in 3.5 wt % NaCl solution at room temperature are reported in Figure 4. The corrosion potential (E_corr_) and the corrosion current densities (I_corr_) were obtained by Tafel’s extrapolation using the CVIEW tools, as were shown in Table 1. The corrosion potential (E_corr_) of SCA solder was −639 mV while the E_corr_ of SCAC and SCAL was −685 mV and −718 mV, respectively. The self-corrosion potential became little more negative after adding Ce and La, it can be explained by the more negative electronegativity of Ce and La. However, the corrosion current density (I_corr_) of SCA decreased from 4.16 × 10^−7^ A·cm^−2^ to 1.43 × 10^−7^ A·cm^−2^ after adding Ce, and from 4.16 × 10^−7^ A·cm^−2^ to 1.19 × 10^−6^ A·cm^−2^ after adding La accordingly. The calculated long-time corrosion rate based on corrosion current density decreased from 0.009 mm/year to 0.004 mm/year. These results indicated that the rare earths Ce and La had a positive effect on the corrosion resistance of SCA solder.

#### 3.2.1. Transformation from Cathodic Polarization to Anodic Polarization

Before the transformation, the dissolved oxygen reduction reaction and hydrogen evolution reaction on the cathode occurred. Then the current density began to increase rapidly at point A, which can be defined as the transformation. At this stage, the cathodic polarization was characterized by dissolved oxygen reduction and hydrogen evolution reaction, which was reported in Gao’s study [8]. The first step was that oxygen dissolved until it was completely consumed

O_2_ + 4e^−^ + H_2_O → 4OH^−^ (2)

Then hydrogen bubbles appeared ceaselessly

2H_2_O + 2e^−^ → H_2_ + 2OH^−^ (3)

It can be seen from the reaction that there was no corrosion products gathering on the cathode, and the surface was observed to be bright at the end of the transformation.

#### 3.2.2. Active/Passive Transition Stage

The active reaction from point A to point B occurred when anodic dissolution took. At this section, the current density continued to escalate dramatically since active Sn dissolved into tin ion. According to Mohran’s study [38], Sn was betatopic as following reaction took place

Sn − 2e^−^ → Sn^2+^ (4)

then Sn^2+^ further oxidized to Sn^4+^

Sn^2+^ − 2e^−^ → Sn^4+^ (5)

**Figure 5:**
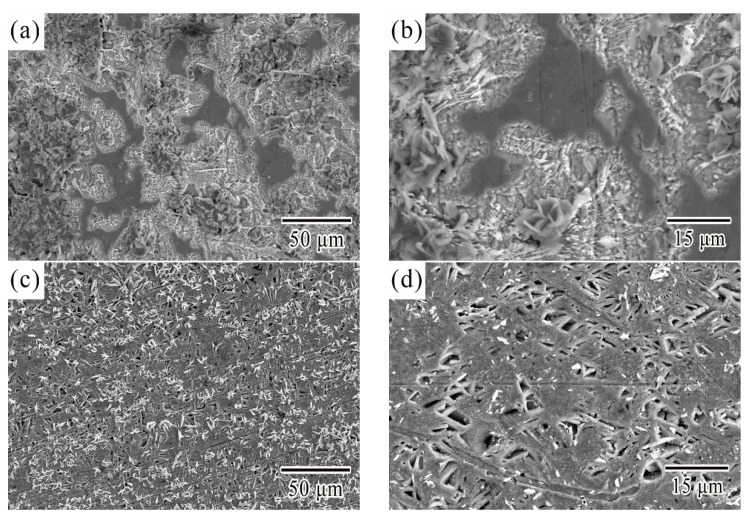

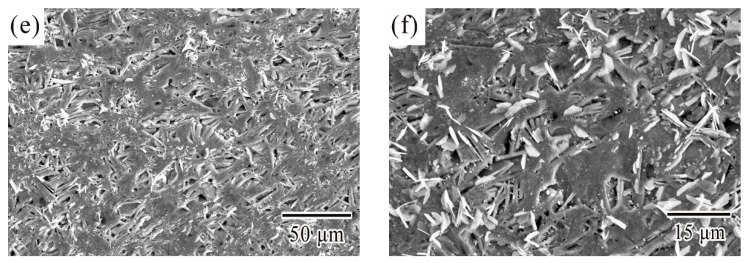
Illustrated the surface morphology of (**a**,**b**) SCA, (**c**,**d**) SCAC, and (**e**,**f**) SCAL solders when the samples polarized up to point B.

After anodic dissolution, the current density tended to decrease from starting over point B. Then the prepassivation section began. The potential and current density at point B was named as the prepassivation potential (E_pp_) and critical current density (I_c_) respectively. A conclusion could be drawn from Table 1 that, although Ce and La addition hardly changed the I_c_ of SCA, the E_pp_ had been effectively reduced which suggested the passivation capability had been improved. Figure 5 showed the corroded surface morphologies of SCA, SCAC, and SCAL solder samples when polarized up around to point B. Figure 5a,b obviously offered an evidence to show the existence of a thin oxide-film that did not completely dissolve. According to the theory of metal passivation, the thin oxide-film should be compact enough to insulate the metal from etching solution mechanically. However, it dissolved fast, and the reason was that it was too thin to overcome the increase of the potential. Once the oxide film broke down, the new uncorroded Sn phase was exposed to the solution, generating an abhurite adsorbing on the surface, especially on the pits of the surface. The adsorbed corrosion products hindered the further corrosion of metals. Thus, the slight reduction of the current density between point B and point C should be the consequence from comprehensive function of multiple factors that mentioned above. Similar effects after adding Ce and La on the surface morphology of SCA could be observed in Figure 5c,e, and the hollows seemed to be the result of dissolved anodic Sn. However, it was interesting that there was no dissolution of oxide film. Moreover, the plate-like corrosion products generated directly on the surface after anodic Sn dissolved. That might explain why SCAC and SCAL solders prepassivated earlier.

#### 3.2.3. Stable Passivation Stage

**Figure 6:**
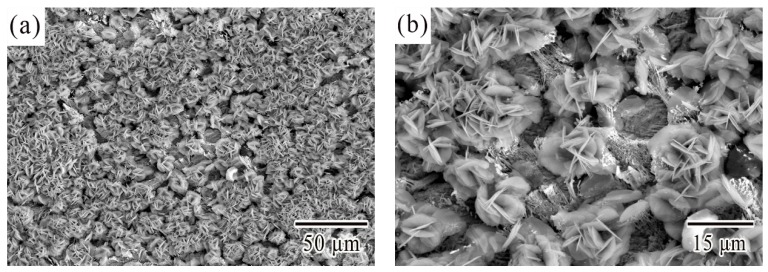

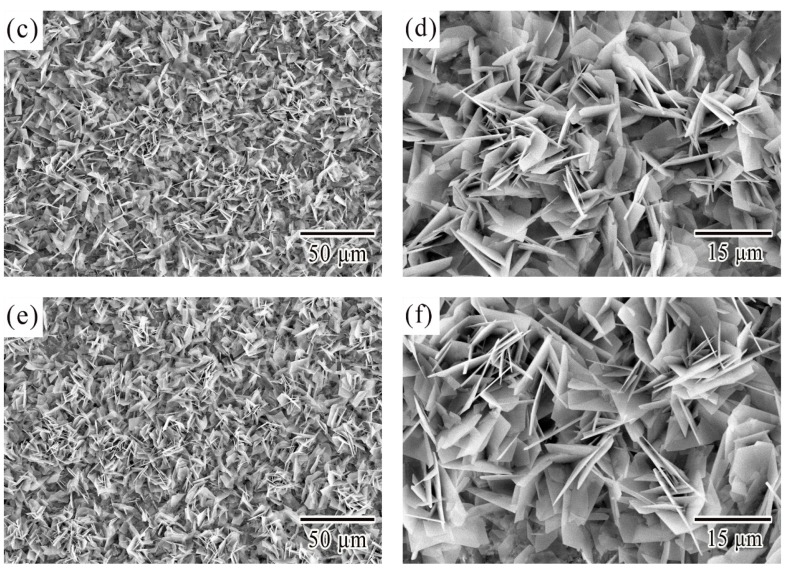
Micrographs of corroded surface of (**a**,**b**) SCA, (**c**,**d**) SCAC, and (**e**,**f**) SCAL solders when polarized to point C.

When the samples polarized up to point C, the passivation state became stable. A trend in Figure 4 showed the current densities that began last lessen were leveling off. At the initial stage of stable passivation, the corrosion products had generated enough to cover the surface of samples, as were shown in Figure 6, and the further corrosion transformed into a harder form that made current density steady-going: corrosive ions had to transfer through corrosion product film to contact unexposed metal.

The average current density from point C to point D was identified as passivation current density (I_p_) in this study. The highest passivation current density (I_p_) 1.40 × 10^−2^ A·cm^−2^ was attained for the sample from SCA solder. By contrast, the I_p_ value of the SCAC and SCAL solders were 0.87 × 10^−2^ A·cm^−2^ and 0.75 × 10^−2^ A·cm^−2^ respectively, about half of the SCA solder’s. Figure 6a illustrated the excessive gap in corrosion product film. The excessive gap enhanced the ion exchange ratio, and the exposed metal still can be seen in Figure 6b. That explained the higher passivation current density (I_p_) of SCA solder. In comparison, the addition of Ce and La contributed to the compact corrosion product film, as was shown in Figure 6c,e, while the exposed metal hardly can be seen in Figure 6d,f.

**Figure 7:**
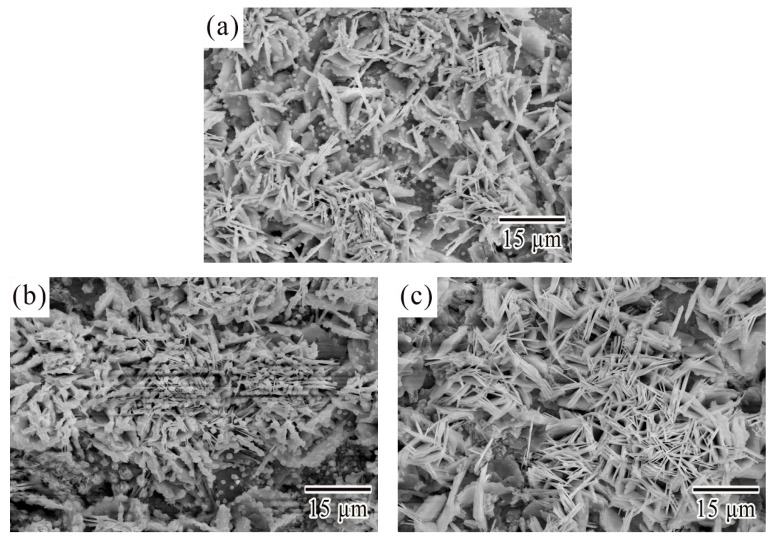
Micrographs of corroded surface of (**a**) SCA, (**b**) SCAC, and (**c**) SCAL solders when polarized to point D.

**Figure 8:**
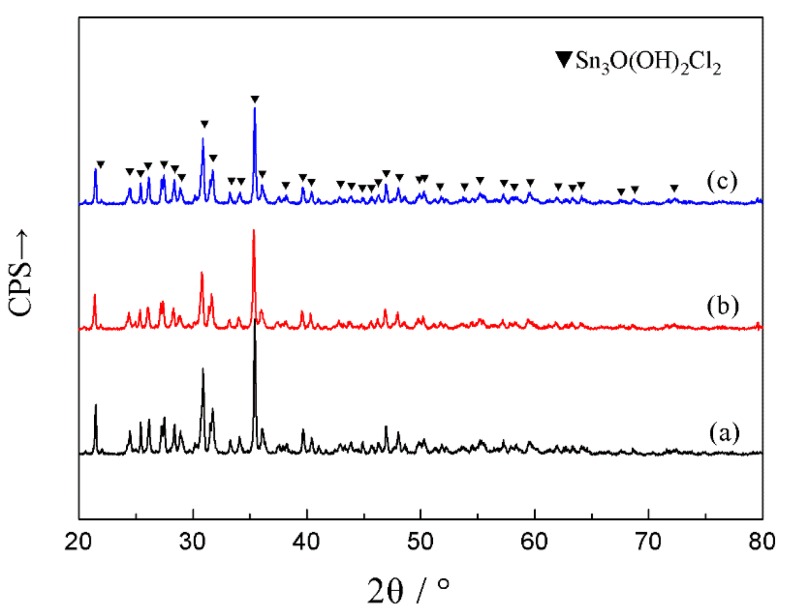
XRD diffraction patterns of corrosion products at surface when polarized to point D: (**a**) SCA, (**b**) SCAC, and (**c**) SCAL solders.

Figure 7 showed the different micrographs of corroded surface after electrochemical potentiodynamic measurements. The results showed that the similar morphology of corrosion products were obtained from SCA, SCAC, and SCAL solders when polarized to point D. The corrosion products were platelet-like shape and were likely to form clusters with different orientations. The XRD patterns for the corrosion products of SCA, SCAC, and SCAL solders after electrochemical potentiodynamic measurements were showed in Figure 8. No peak of pure Sn was detected, which confirmed that the surface of solders was full of corrosion products. The dominant peaks were retrieved, and the corrosion products were proved to be Sn_3_O(OH)_2_Cl_2_. The equation gives an interpretation to the formation of this tin oxy-hydroxide chloride that had already been reported [8,30]

3Sn + 4OH^−^ + 2Cl^−^ → Sn_3_O(OH)_2_Cl_2_ + H_2_O (6)

**Figure 9:**
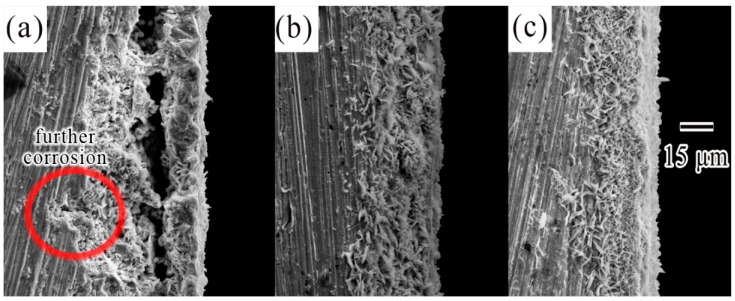
SEM images obtained from the cross section of (**a**) SCA, (**b**) SCAC, and (**c**) SCAL solders after tests.

In order to reveal the differences between SCA solder and rare earth-adding solders from a macroscopic view, the cross sections of these solders had been captured in Figure 9. In Figure 9a, on one hand, the structure of corrosion product film was separated into two parts, and thus the formative gap would recede the adhesion strength of the outer part. On the other hand, corrosive chlorium that transferred through the looser structure would cause further corrosion, which was shown by red encircled regions. However, with the addition of Ce and La (see Figure 9b,c), the structure of corrosion product film was integral, and the film tightly adhered to the surface. This indicated a greater passivation property in SCAC and SCAL solders, with the SCAL solder being best. The corresponding result was found in literature [39], which said that the CeO_2_ in Sn-Ag solder brought in a more adherent, compact, and finer sheet-like structure of corrosion products which created a better passivation property.

**Figure 10:**
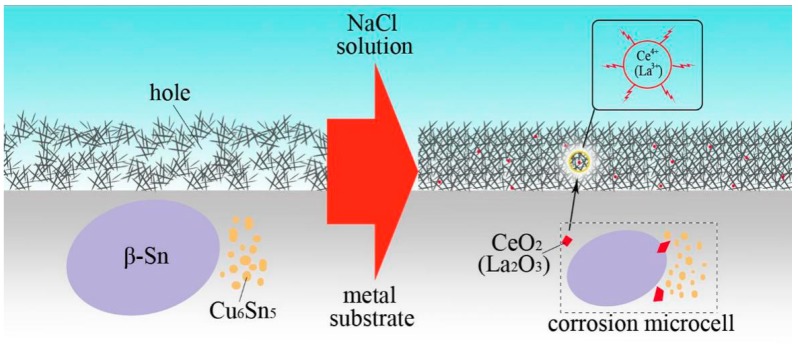
Schematic diagram of CeO_2_ (La_2_O_3_) inhibiting the galvanic corrosion and Ce^4+^ (La^3+^) ions restraining the hole of corrosion products.

A schematic diagram was depicted to illustrate the action mechanism of better corrosion resistance and better passivation property of SCAC and SCAL solders, as shown in Figure 10. Abundant corrosion microcells exist in the metal substrate. Every microcell is favorable with β-Sn serving as cathode and Cu_6_Sn_5_ as anode. As mentioned above, the finer β-Sn grains and homogeneously distributed Cu_6_Sn_5_ particles were formed concurrently, intermingled with the CeO_2_ (La_2_O_3_). As a result, the cathode/anode area ratio of the electrochemical cell is reduced, thus the corrosion rate of galvanic corrosion is inhibited. Furthermore, since the standard potential of CeO_2_ (La_2_O_3_) is more positive than β-Sn, a fraction of Ce^4+^ (La^3+^) ions will release from CeO_2_ (La_2_O_3_) particles into the solution [40]. Originally, the platelet-like shape corrosion products Sn_3_O(OH)_2_Cl_2_ are inclined to cluster together on the metal substrate surface, these clusters are relatively free to develop in the NaCl solution environment, and finally cause the hole forming in the gap of the clusters. However, with the Ce^4+^ (La^3+^) ions dissociated in the gaps, their strong absorptive capacity restrains the vacancy condensation of the clusters. Due to the Ce^4+^ (La^3+^) ions acting as the surface modifier, the structure of corrosion products become adherent and compact, which effectively prevents the Cl^−^ from passing through the product layer to further corrode the material [41,42].

## 4. Conclusions

The microstrcture of Sn-0.7Cu-0.075Al solder alloy consisted of β-Sn and Cu_6_Sn_5_/Sn eutectic, and Al_2_Cu was not found with the addtion of Al, the trace Al atoms may have solid soluted in the Sn matrix. With the addtion of Ce and La, the square-like CeO_2_ and La_2_O_3_ phases were generated dispersively, which lead to the refined grains of Sn-0.7Cu-0.075Al solder alloy. Furthermore, a directional tendency of grain growth emerged.

The self-corrosion potential of Sn-0.7Cu-0.075Al solder alloy became little more negative than before after adding Ce and La. However, the corrosion current density (I_corr_) values of Ce (La)-containing solders decreased up about three times. The phenomenon that a more adherent, compact and finer sheet-like structure of corrosion products forms after adding Ce and La should be noticed. By contrast, the corrosion product film of Sn-0.7Cu-0.075Al was coarse and loosely adherent to the surface, which lead to passivation current density (Ip) reduced by half at the stage of passivation. All the evidence indicated that a better corrosion property showed up with the addition of Ce and La in Sn-0.7Cu-0.075Al solder alloy, with the La-containing solder being best.

## Figures and Tables

**Table 1:** Electrochemical parameters of SCA, SCAC, and SCAL solders in 3.5% NaCl solution

Material	E_corr_ (mV)	I_corr_ (A·cm^−2^)	E_pp_ (mV)	I_c_ (A·cm^−2^)	E_p_ (mV)	I_p_ (A·cm^−2^)	Corrosion Rate (mm/a)
SCA	−639	4.16 × 10^−7^	113	7.69 × 10^−2^	334	1.40 × 10^−2^	0.009
SCAC	−685	1.43 × 10^−7^	71	7.76 × 10^−2^	344	0.87 × 10^−2^	0.004
SCAL	−718	1.19 × 10^−7^	84	7.41 × 10^−2^	357	0.75 × 10^−2^	0.004
